# Absence of covert face valuation in Autism

**DOI:** 10.1038/s41398-021-01551-z

**Published:** 2021-09-07

**Authors:** Fabien Vinckier, Mathias Pessiglione, Baudouin Forgeot d’Arc

**Affiliations:** 1 Université de Paris, F-75006 Paris, France; 2 Department of Psychiatry, Service Hospitalo-Universitaire, GHU Paris Psychiatrie & Neurosciences, F-75014 Paris, France; 3 Motivation, Brain & Behavior (MBB) lab, Paris Brain Institute (ICM), Hôpital Pitié-Salpêtrière, F-75013 Paris, France; 4 Sorbonne University, Inserm, CNRS, Paris, France; 5 CHU Sainte-Justine Research Center, Montréal, QC H3T 1C5 Canada; 6 Department of Psychiatry, Université de Montréal, Montréal, QC H3C 3J7 Canada; 7 Centre intégré Universitaire du Nord-de-l’Île-de-Montréal, Montréal, QC H1E 1A4 Canada

**Keywords:** Human behaviour, Autism spectrum disorders

## Abstract

Autism is a neurodevelopmental condition defined on clinical criteria related to diminished social reciprocity and stereotyped behavior. An influential view explains autism as a social motivation disorder characterized by less attention paid to the social environment and less pleasure experienced with social rewards. However, experimental attempts to validate this theory, by testing the impact of social reward on behavioral choice and brain activity, has yielded mixed results, possibly due to variations in how explicit instructions were about task goals. Here, we specified the putative motivation deficit as an absence of spontaneous valuation in the social domain, unexplained by inattention and correctible by explicit instruction. Since such deficit cannot be assessed with behavioral measures, we used functional neuroimaging (fMRI) to readout covert subjective values, assigned to social and nonsocial stimuli (faces and objects), either explicitly asked to participants (during a likeability judgment task) or not (during age or size estimation tasks). Value-related neural activity observed for objects, or for faces under explicit instructions, was very similar in autistic and control participants, with an activation peak in the ventromedial prefrontal cortex (vmPFC), known as a key node of the brain valuation system. The only difference observed in autistic participants was an absence of the spontaneous valuation normally triggered by faces, even when they were attended for age estimation. Our findings, therefore, suggest that in autism, social stimuli might fail to trigger the automatic activation of the brain valuation system.

## Introduction

Autism[1, 2]^1^ refers to a broad spectrum of neurodevelopmental conditions that manifest as altered social interaction, atypical communication along with increased reactivity to the sensory environment, and stereotyped behaviors. Autism has an estimated prevalence of about 1% worldwide and represents a major public health issue [3]. Nevertheless, socio-cognitive alteration, that is arguably the cornerstone of autism, remains poorly understood. An influent theory suggests that a major explanatory factor is a lack of social motivation [4]: autistic persons tend to report less pleasure in social activities, and spend less resources seeking social rewards. Yet there is still debate on how social interactions in autism should be described and whether they are better interpreted in terms of atypical motivation, rather than disabilities in cognitive functions such as perception, mentalizing, or executive control [5].

Most of the experimental investigations of the motivation deficit have focused on the neural responses to social rewards in autistic persons. Individual preferences can be related to neural activity in a set of brain regions, termed brain valuation system (BVS), including the ventromedial prefrontal cortex (vmPFC) as a central component [6–9]. The BVS is able to assign values to items from different categories, enabling their comparison on a common scale [10, 11]. This neural value signal is therefore believed to underpin preferences between options that are expressed in choice behavior [11, 12]. The expectation was therefore that in autistic persons, because low values would be assigned to social rewards, BVS activity would be lower than in neurotypical persons. Yet the results have been somewhat inconsistent: some confirm a lack of response to social rewards [13–15], while others report an equivalent or even more pronounced deficit in processing nonsocial rewards such as money [16, 17] (see [18] for a recent meta-analysis). These conflicting results might relate to the type of stimuli used during fMRI scanning [19], and to their ecological relevance in particular, but also to the instructions being more or less explicit about the goal to maximize reward in the tasks performed. Indeed, explicit instructions can modulate social information processing in autistic persons. For instance, autistic persons can pass the false belief task (used to assess the theory of mind) when explicitly asked to do so, even if they do not show evidence of spontaneous belief attribution [20, 21]. Similarly, they can process facial expression, tone of voice, or gaze direction in accordance with instructions, even if they spontaneously ignore these features during natural conversations [21, 22]. Building on this dissociation, we reasoned that autistic persons may not spontaneously monitor the value of social stimuli, but would be able to do so following explicit instructions. This behavior could still be viewed as a social motivation deficit, since the ability to valuate social stimuli would be preserved, even if not employed spontaneously. Furthermore, the absence of spontaneous valuation could be a reason for (and not a consequence of) autistic persons paying little attention to social cues, which implies that it could be observed even for social stimuli that are attended for independent purposes.

This conception of the social motivation deficit can hardly be tested on behavioral responses, because it needs accessing subjective values without asking them to participants. We, therefore, turned to fMRI and capitalized on a critical functional property of the BVS: its automaticity—meaning that neural value signals are covertly generated, even when the ongoing task does not involve any valuation process. The only requirement is that the item to be valued must be attended, which can be obtained with orthogonal instructions to perform a task involving no valuation process, such as judging an independent attribute (e.g., the age of the person in the case of faces) [23, 24]. Thus, our key prediction regarding fMRI data was that, contrary to neurotypical persons, autistic persons would not show any covert value-related activity in their vmPFC, while busy judging the age of persons from their faces. In contrast, they would show normal overt value-related activity when explicitly instructed to judge how much they like the faces. For this pattern to qualify as specific to social motivation, it should not be observed with stimuli from a nonsocial domain such as objects, which should be assigned values in both the tasks with orthogonal instructions (size estimation) and explicit instructions (likeability judgment).

To test these predictions, we recruited two matched groups of 19 autistic participants and 19 control participants (see details in Table 1). During fMRI scanning, they performed both valuation and estimation tasks, on both faces and objects. To assess whether likeability ratings were reliable measures of subjective valuation, we tested their consistence with a preference between objects and confidence ratings about likeability, in two post-scan sessions. Confidence in (explicit) judgments is also supposed to be automatically computed by the BVS [25]. Our main prediction was a three-way interaction (group × domain × task) in value-related vmPFC activity, driven by a specific difference between autistic and control participants when judging the age of faces. A secondary hypothesis was that values assigned to faces might be less reliable in autistic participants. We assessed the reliability of likeability ratings in post-scanning sessions, first by testing whether they would predict binary choices (preferences between two items), second by measuring their stability when probed again 1 month later, and third by asking for confidence (second-order ratings) about likeability judgments.

**Table 1:** Demographic and psychometric details.

	Autistic participants *N* = 19	Control participants *N* = 19	*p* value
Sex (W/M)	6 / 13	4 / 15	0.157
Age (years)	26.8 (8.4)	24.3 (4.3)	0.258
IQ	107.3 (13.8)	106.9 (11.9)	0.920
ADOS communication	4.8 (1.6)		
ADOS social	9.9 (2.3)		
ADOS imagination	1.3 (0.8)		
ADOS total	16.0 (3.6)		

Data are provided as mean (std).

## Methods and materials

### Participants

Nineteen adults with autism and 19 controls were included in the study. Intelligence Quotients were determined by the Wechsler Adult Intelligence scale. No significant differences in age and IQ were found between groups. Participants’ demographic characteristics are summarized in Table 1. All participants in the autism group were recruited from the diagnostic clinic at Hôpital Rivière-des-Prairies, Montréal, Canada. All had been diagnosed by expert clinicians on the basis of DSM-IV criteria, using standardized instruments [26, 27]. Control participants were recruited through advertisements. One participant in the control group was excluded because of technical issues, and another one completed only the first visit.

### Ethics statements

This research was performed in accordance with institutional ethical guidelines, which comply with the guidelines of the declaration of Helsinki. The research protocol was approved by the Ethical Committee of the Hôpital Rivière-des-Prairies, Montréal (CER HRDP 14-13), and Regroupement Neuroimagerie Québec (CMER RNQ 14-15-014). All participants signed informed consent forms before beginning the study.

### Stimuli

We used 168 faces and 168 objects images. Faces were drawn from the Productive Aging Lab Face Database [28] and from FACES Database [29]. They were selected to cover a large range of ages (19–75 years), different eye and hair colors, as well as both genders. They had a neutral facial expression, to avoid trivial stimulus-driven valuation, as would be obtained for instance with smiling faces. Objects were drawn from the Boss database [30]. They were chosen to cover a large variety of preferences. In a pilot behavioral study, 12 participants rated the estimated price of the 168 objects. Object images were square while face images were in portrait format (ratio = 1.25). Images were sized to cover one third of the screen in width dimension. On average, luminance was higher for objects (207) than for faces (127). To compare explicit and orthogonal instructions, the 168 images of each category were split into two sub-lists of 84 images, which were matched in terms of luminance and content (age and gender for faces, prices for objects).

### Design

Each participant was required to come to the lab twice, 1 month apart (median interval of 4.3 weeks between the two visits).

#### First visit

**Fig. 1:**
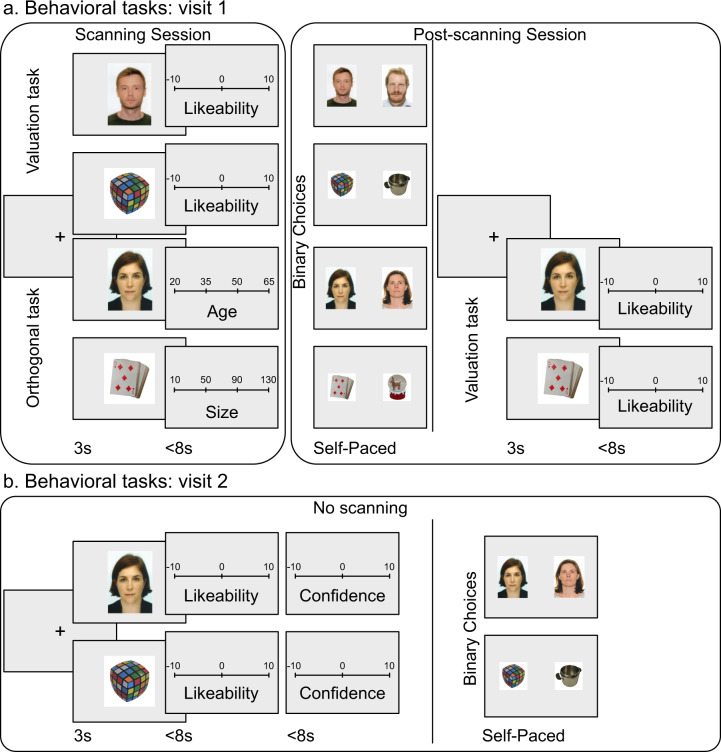
Behavioral tasks. **a** Visit 1. During the scanning session (left), participants performed the valuation task (likeability rating) on half the items and an orthogonal task (age rating for faces and size rating for objects) on the other half. Each of the four blocks comprised 84 items in a randomized order. The order of the four blocks was randomized as well. After the scanning session (right), participants performed a series of 336 choices between two stimuli of the same category. Faces were paired by gender. Objects were paired such that the prices of the two items was approximatively matched. **b** Visit 2. The second visit took place around 4 weeks later and involved no scanning. Participants performed first the valuation task, augmented with a second-order rating (of how confident they were in their likeability rating), on all the items. Then, they were required to make the same series of choices as in the first visit, in the same order.

After a short training session that was performed in a mock scanner, pictures representing faces or objects were presented one by one in the MRI scanner, and participants were asked to rate either the likeability (valuation task)—for half of the stimuli of each category—or an orthogonal attribute of the stimulus (orthogonal task)—for the other half (see Fig. 1). The attribute that participants rated during the orthogonal task was the age for faces (in year) or the size (line with maximal length) for objects (in cm or inches depending on participant choice). Half the participants in each group performed the orthogonal task first and then the valuation task, whereas the other half followed the reverse order. In each of these subgroups, half the participants began with faces and half with objects. The sub-lists of images used in each task were also pseudo-randomized across participants. This procedure has the advantage of canceling out order effects while preserving the possibility of scanning the orthogonal task before participants heard about the (explicit) likeability rating and choice tasks.

At the trial level, a similar rating procedure was implemented both for the orthogonal and valuation tasks. The picture was displayed on the screen for 3 s, following a fixation cross. Then appeared the rating scale, graduated between −10 (not at all) and 10 (tremendously) for likeability rating, between 20 and 65 years for age rating (faces), and between 10 and 130 cm (4″ to 50″) for size rating (object). In all cases, the scale had 21 steps (10 values on each side of the center), but only three or four reference graduations were shown. Participants could move the cursor by pressing a button with the right index finger to go left or with the right middle finger to go right. The rating was self-paced (up to 8 s) and participants had to press a button with the left index finger to validate their responses and go to the next trial. The initial position of the cursor on the scale was randomized to avoid confounding the ratings with the movements they involved.

After the scanning sessions, participants were first asked to make a series of 336 choices between two stimuli of the same category and the same sub-list. Faces were paired by gender. Objects were paired such that the prices of the two items were approximatively matched. Face and object pairs were intermixed in a pseudo-randomized order. At the trial level, after a fixation cross, the two items were presented simultaneously, one on each side of the screen. The choice was self-paced, and participants chose by pressing the left or the right arrow of the keyboard. Then, participants were required to rate the likeability of the stimuli used in the orthogonal task, following the same procedure as inside the scanner.

#### Second visit

During the second visit, participants were first required to perform the valuation task again for all images. The procedure and the order of images was the same as in the first visit, except that after the likeability rating, they also had to rate how confident they were in their rating, on a scale between −10 (not at all) and 10 (fully). Then, they were required to make the same series of choices than in the first visit, in the same order. Please note that as the orthogonal tasks were not performed in the second visit, confidence estimates were not collected for age/size ratings.

All tasks were programmed using the Matlab and Psychtoolbox 3.0.11 [31] (http://psychtoolbox.org/).

### Behavioral analyses

#### Likeability and orthogonal ratings

Average and standard deviation (SD) of ratings were computed for each participant and each domain (faces/objects) and entered in ANOVAs with the domain as within-subject factor, group as between-subject factor, and subject as a random factor. We also assessed the stability of ratings using two measures: averaged item-wise absolute difference between the two visits, and Pearson’s correlation coefficient between first and second rating. Both measures were computed for each participant and each domain and entered in ANOVAs with a domain as within-subject factor, group as between-subject factor, and subject as a random factor.

#### Binary choices

The proportion of predicted choices (i.e., likeability rating(A) ≥ likeability rating(B) and A chosen over B) was computed for each participant and each domain (faces/objects) and entered in ANOVAs with a domain as within-subject factor, a group as between-subject factor, and subject as a random factor. We also performed logistic regressions. For each participant and each domain, binary choices were regressed against rating difference as a regressor, and regression coefficient were entered in ANOVAs with a domain as within-subject factor, group as between-subject factor, and subject as a random factor. Finally, we assessed the stability of choices with the proportion of consistent choice, that was computed for each participant and each domain and entered in ANOVAs with a domain as within-subject factor, group as between-subject factor, and subject as a random factor.

#### Confidence ratings

Average ratings were computed for each participant and each domain (faces/objects) and entered in ANOVAs with domain as within-subject factor, group as between-subject factor, and subject as random factor. We also investigated the link between likeability and confidence using a polynomial regression. For each participant and each domain, confidence ratings were regressed against *z*-scored likeability ratings and squared *z*-scored likeability ratings. Coefficients of regression were entered in ANOVAs with a domain as within-subject factor, group as between-subject factor, and subject as a random factor.

### fMRI analyses

T2*-weighted echo planar images (EPIs) were acquired with BOLD contrast on a 3.0 Tesla magnetic resonance scanner. We employed a tilted plane acquisition sequence designed to optimize functional sensitivity in the OFC. To cover the whole brain with a good temporal resolution, we used the following parameters: TR = 2.29 s, 35 slices, 2 mm slice thickness, 1 mm interslice gap. T1-weighted structural images were also acquired, coregistered with the mean EPI, segmented, and normalized to a standard T1 template to allow group-level anatomical localization. All data processing and analysis was done using statistical parametric mapping software SPM12 [32] (https://www.fil.ion.ucl.ac.uk/spm/) implemented in Matlab. Preprocessing consisted of spatial realignment, normalization using the same transformation as structural images, and spatial smoothing using a Gaussian kernel with a full-width at half-maximum (FWHM) of 8 mm.

Preprocessed individual time series in each voxel were regressed against the following GLMs. All GLMs included two categorical regressors, at an image and at scale onsets. In the first GLM, the image-onset regressor was parametrically modulated by *z*-scored likeability rating (provided either inside or outside the scanner, but always during the first visit). In the second GLM, the sessions corresponding to the valuation task were similar to the first GLM, while for the sessions corresponding to the orthogonal task, the image-onset regressor was parametrically modulated both by *z*-scored orthogonal rating and *z*-scored likeability rating provided during the first visit (in that order). In the third GLM, the image-onset regressor was parametrically modulated both by *z*-scored orthogonal rating and *z*-scored confidence rating, provided during the second visit. All regressors were convolved with the canonical hemodynamic response function of SPM12 with its first-order derivative. To correct for motion artifacts, participant-specific realignment parameters were modeled as covariates of no interest. Regression coefficients were estimated at the individual level using the restricted maximum-likelihood estimation. Linear contrasts of regression coefficients were computed at the participant level and then taken to group-level random effect analyses, using one-sample *t*-tests. Statistical maps were family-wise error corrected for multiple comparisons at the cluster level. Whole-brain results were visualized using the xjView toolbox (http://www.alivelearn.net/xjview).

We further analyzed the ROI defined by the conjunction of the linear activation with likeability rating in the two tasks (voxel-generating threshold *p* < 0.01 uncorrected), pooling both groups and both domains, within a vmPFC mask from the literature [8]. Regression estimates (β coefficients) of the linear activation with likeability rating were extracted for each participant and each of the four conditions and entered in random-effect group-level analyses.

## Results

### Behavior

During fMRI scanning (first session of visit 1 in Fig. 1), items were presented one by one on a computer screen, followed by a rating scale. Half the items were presented in the valuation task and the other half in the orthogonal task. In the valuation task, participants rated the likeability of both faces and objects, whereas, in the orthogonal task, they rated either the age (of faces) or the size (of objects). In order to know the subjective values that participants would assign to the items presented in the orthogonal task, all these items were shown again one by one in a post-scanning session (second session of visit 1 in Fig. 1), using a valuation task analogous to that performed in the fMRI scanner.

**Fig. 2:**
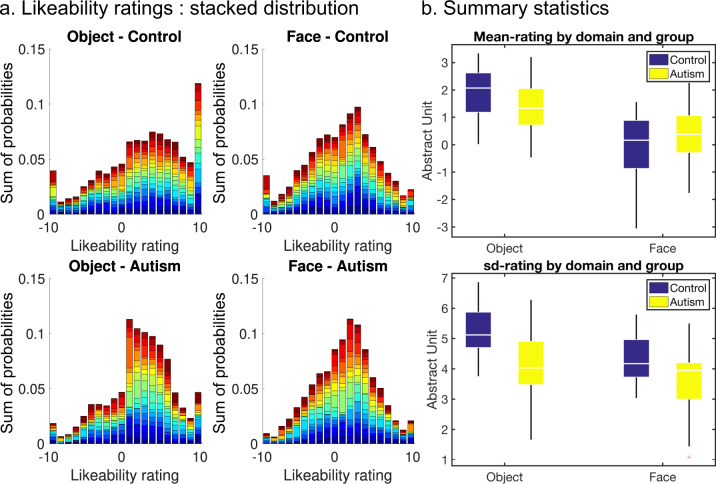
Likeability ratings. **a** Individual data. Panels show stacked histograms of likeability ratings for both groups (top: control participants, bottom: autistic participants) and both domains (left: objects, right: faces). In both groups, each participant is represented with a different (arbitrary) color. For each group and domain, data were normalized such that the grand sum of all bins and all participants is equal to 1. **b** Summary group-level statistics. Top panel shows the mean, while the bottom panel shows the standard deviation of likeability rating (across items). White line: median, points are drawn as outliers if they are larger than Q3 + 1.5 × (Q3 − Q1) or smaller than Q1-1.5 × (Q3 − Q1), where Q1 and Q3 are the 25th and 75th percentiles, respectively. Vertical error bars are inter-participant s.e.m.

When comparing the distribution of likeability ratings (Fig. 2a), the most striking feature observed in autistic participants was a lower variance, related to control participants giving more extreme ratings (reaching the bounds of the scale). This is formally shown by the ANOVA performed on rating SD, with a main effect of the group [*F*(1, 35) = 7.9, *p* = 0.008] and a significant interaction between group and domain [*F*(1, 35) = 5.0, *p* = 0.032], due to SD reduction in autistic participants being more pronounced with objects than with faces (Fig. 2b). The same ANOVA performed on mean ratings revealed no group effect, but a significant interaction [*F*(1, 35) = 7.8, *p* = 0.008], yet opposite to the prior idea that autistic persons would value the objects more and the faces less than neurotypical persons (Fig. 2b). Note that we do not comment on the main effect of the domain, as it might depend on the particular selection of faces and objects in our material. Finally, the response time (RT) tended to be longer in autistic participants, but the difference with control participants was not significant (*p* = 0.132).

Regarding orthogonal ratings (age and size estimates), there was no group effect (*p* = 0.952) and no group-by-domain interaction (*p* = 0.263). Again, we do not interpret the main effect of the domain, as comparing ages and sizes would be meaningless. Importantly, the accuracy of age ratings was similar in both groups. Indeed, the absolute difference between the actual age of the persons and the age estimate was similar in both groups (6.4 years in control participants vs. 6.5 years in autistic participants, *p* = 0.788). This is an important control as it means that autistic participants actually paid attention to faces during the orthogonal task. Note that we cannot report the same analysis for objects as we don’t have the actual size of objects. Autistic participants also tended to be slower than control participants, but the difference in RT was not significant (*p* = 0.223). We tested the independence of value and orthogonal ratings: there was a significant negative correlation between value and age ratings (β = −0.38, *p* < 0.001) and a significant positive correlation between value and size ratings (β = 0.13, *p* < 0.001). We, therefore, corrected for orthogonal ratings when assessing the link between value ratings and brain activity.

**Fig. 3:**
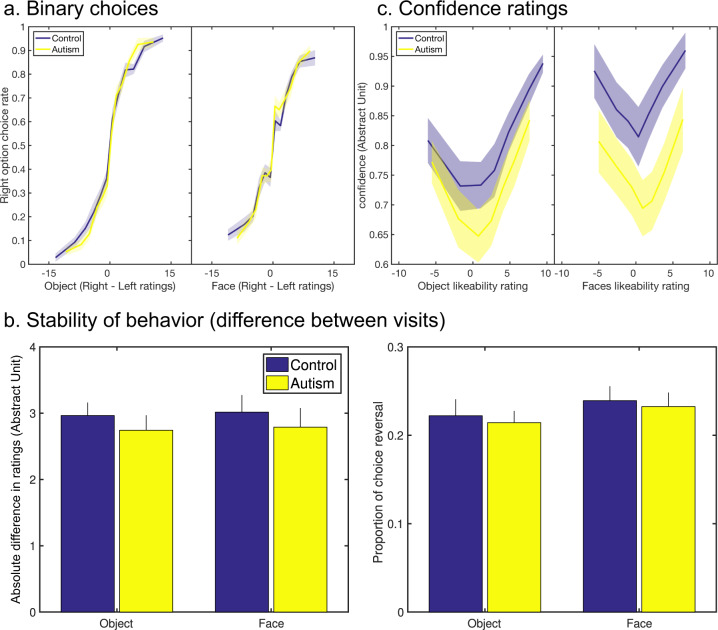
Relations between likeability ratings and other behavioral variables. **a** Binary choices. Probability to choose the right object/face over the left one as a function of the difference between likeability ratings (obtained in the first visit). **b** Stability of behavior. The absolute difference between visits for likeability ratings (left panel) and proportion of choice reversal for binary choices (right panel). **c** Confidence ratings as a function of likeability ratings (obtained in the second visit). Shaded areas and error bars represent inter-participant s.e.m. In every panel, data were plotted on the left for objects and on the right for faces, in blue for control participants and yellow for autistic participants.

One important question to ensure comparability of BVS activity between autistic and control participants is that likeability ratings are equally reliable. We, therefore, tested whether these ratings could predict other behavioral measures. During the post-scanning session, we had participants perform a binary choice task in which items were presented in pairs, with the instruction to indicate their subjective preference (see Fig. 1). Choices were accurately predicted by likeability ratings (A should be preferred to B if rated higher than B) in autistic and control participants (mean prediction score: 79.5%, see Fig. 3a), with no effect of group (*p* = 0.947) or group × domain interaction (*p* = 0.686). The same (no group effect and no interaction) was found when comparing beta estimates of logistic regression of choices against ratings.

To further ensure that ratings could predict preferences, we had participants back to the lab 1 month (33.2 days on average, SD = 11.2 days) after the fMRI experiment (visit 2 in Fig. 1), and perform the same tasks as during the post-scanning session of visit 1. We found no group effect or group-by-domain interaction in any of our stability measures (Fig. 3b): delta-rating (item-wise difference between the two visits, *p* = 0.492 and *p* = 0.990 for the main effect of group and group-by-domain interaction, respectively), Pearson’s correlation coefficients between first and second rating across items (with a main effect of domain: mean: *r* = 0.66 for faces and *r* = 0.63 for objects; *p* = 0.001 but no main effect of the group; *p* = 0.515 and no group-by-domain interaction; *p* = 0.527), or proportion of consistent choice across visits (mean: 77%; *p* = 0.710 and *p* = 0.965 for the main effect of group and group-by-domain interaction, respectively). The plots may give the impression of stronger stability of ratings in autistic participants, but this is due to less variable ratings in the first place. Indeed, the numerical trend was reversed (although still not significant) when comparing *z*-scored ratings.

Finally, we added a confidence rating task, on top of likeability rating, in the post-scanning session (i.e., participants indicated their confidence in their rating). Please note that there is no normative correct answer in likeability rating and therefore no notion of metacognitive accuracy in confidence rating. Autistic participants tended to be less confident than control participants (*F*(1, 34) = 3.6, *p* = 0.068), and there was a main effect of domain with higher confidence for faces (*F*(1, 34) = 5.3, *p* = 0.028) but no group × domain interaction (*p* = 0.357). First-order (likeability) ratings are known to predict second-order (confidence) ratings through a quadratic function [33]. Consistently, the quadratic relationship between value and confidence was significant here in both control and autistic participants (both *p* < 0.001), with no group effect (*p* = 0.634) or group × domain interaction in the quadratic regression estimate (*p* = 0.711) (Fig. 3c).

Thus, behavioral measures suggested that likeability ratings were reliable measures of item subjective values in both groups, being significant predictors of both confidence and choice, and showing similar stability over a 1-month delay.

### fMRI: value signals

#### Whole-brain analysis

**Fig. 4:**
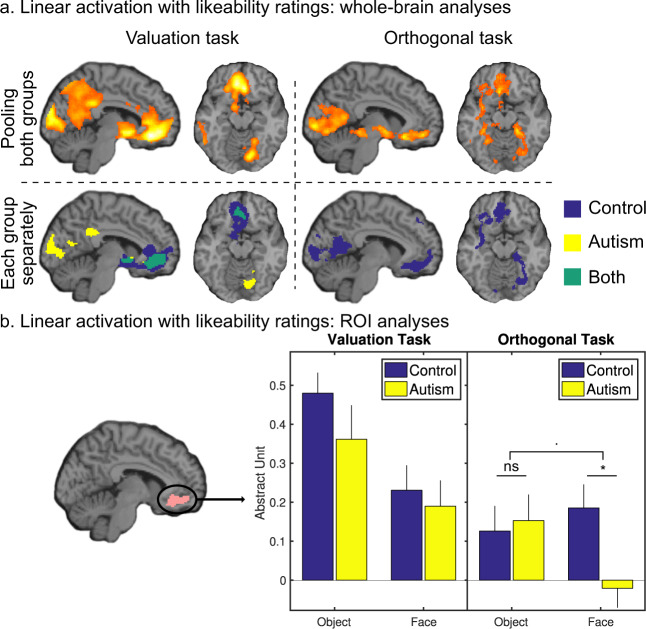
Neural value signals. **a** Whole-brain analysis. Statistical Parametric Maps show linear activation with likeability ratings, pooling all participants together (top panels) or in each group separately (bottom panels—blue: control participants, yellow: autistic participants, green: both groups). Note that likeability ratings were expressed during scanning for the valuation task (left panels) and after scanning for the orthogonal task (right panels). **b** ROI analysis focusing on the vmPFC (left map). Likeability regression estimates were extracted during the valuation task (left barplot) and during the orthogonal task (right barplot) for each participant and then compared between tasks and groups. Error bars represent inter-participant s.e.m.

To identify the BVS, we regressed a GLM including likeability rating against fMRI activity at item onset, pooling across groups and domains. During the valuation task, significant regression estimates (*p* < 0.05, after FWE correction for multiple comparisons) were found in canonical BVS regions (i.e., vmPFC, ventral striatum, and posterior cingulate cortex), as well as in bilateral primary visual cortex (Fig. 4a). The pattern of value-related brain activity was similar in autistic and control participants, with no significant difference between groups. During the orthogonal task, some linear activation with likeability rating (collected after scanning) was also observed in the same BVS regions, but with lower statistics (even if surviving FWE correction). The pattern of activation was unchanged when including orthogonal rating as a covariant regressor in the GLM, meaning that covert value signals observed during the orthogonal task were not due to the correlation with age or size estimates. Even if value signals were globally lower in autistic participants during the orthogonal task, there was no significant difference between groups. Finally, similar results were observed, with a stronger correlation during the valuation relative to the orthogonal task, when using likeability ratings collected during the second visit (1 month later). This control analysis confirms that the difference was due to the task and not to time (i.e., to a potential change in value between scanning and post-scanning sessions).

#### ROI analysis

In order to better specify the difference between valuation and orthogonal tasks, and test our working hypothesis, we defined an ROI by the conjunction of value-signaling clusters in the two tasks at a permissive threshold (*p* < 0.01, uncorrected), within a vmPFC mask, independently defined from the literature [8]. Likeability regression estimates were extracted for each participant and compared between domains, tasks, and groups (Fig. 4b). When including orthogonal ratings in the GLM, the three-way (group × domain × task) interaction in vmPFC value signals was marginally significant [*F*(1, 35) = 3.9; *p* = 0.056]. The three-way interaction was due to the two-way (group × domain) interaction being significant during the orthogonal task [*F*(1, 35) = 4.7; *p* = 0.038], but not during the valuation task (*p* = 0.549). The two-way interaction itself was due to the difference between groups being significant for faces (*T*(35) = 2.8, *p* = 0.009) but not for objects (*p* = 0.842). This presence of covert value signals in control participants (but not in autistic participants) for faces during the orthogonal task was the only significant difference between groups.

### fMRI: confidence signals

We also explored the neural correlates of confidence, with a GLM that included both likeability and confidence ratings collected during the second visit. Please note that it is the confidence about likeability that was used in both tasks (confidence ratings about age/size ratings were not collected). Thus, the rationale behind this analysis was to investigate whether second-order estimates (i.e., overt and covert confidence) are automatically computed or not, when valuation is explicitly instructed versus automatically engaged. During the valuation task, significant confidence regression estimates were observed in the BVS, although the activation peak in the medial prefrontal cortex was slightly more dorsal than that obtained with subjective value (Fig. S1a). Again, the pattern of activation was similar in autistic participants and control participants, without significant differences between groups in the ROI analysis (Fig. S1b). During the orthogonal task, there was no significant correlate of confidence in brain activity, which is consistent with the idea that confidence is automatically computed for a response that is overtly provided by the participant (i.e., likeability rating during the valuation task) but not for a response that is covertly generated (i.e., likeability judgment during orthogonal tasks).

## Discussion

Our study explored overt and covert valuation of social and nonsocial stimuli, in autistic versus control participants. The only difference was found in the covert valuation of social stimuli: while controls automatically judge the likeability of faces, autistic participants would not spontaneously engage in such a valuation process. However, autistic participants do assign values to faces when explicitly asked, and spontaneously valuate nonsocial stimuli such as objects. These findings have important implications for our understanding of autism, as they uncover a specific form of altered social motivation.

Autistic participants’ brain activities and behavioral responses were similar to controls in many respects. At a behavioral level, likeability ratings assigned to both objects and faces were equally predictive of choice and confidence, and equally stable across time. At the brain level, likeability ratings were represented in a similar BVS, with an activation peak in the vmPFC. These findings argue against a general motivation deficit affecting the valuation process itself, as suggested in some previous studies [17, 34, 35]. Such a general account would predict an alteration of reward processing in autism, which would result in incentive motivation and reward learning deficits at the behavioral level, and to reduced response to rewards at the brain level, irrespective of the type of stimuli and task used. Although some studies reported an impairment of reward maximization, these results remain open to other interpretations: for instance, some rewards like money might be simply less attractive for autistic individuals. Here, we escaped this potential problem by explicitly assessing the subjective value of our stimuli and checking that autistic participants were as interested as control participants. Another factor relates to instructions, which might not have been explicit enough in previous studies for autistic individuals to trigger valuation processes. Here, we specifically controlled for this aspect by contrasting explicit and orthogonal instructions.

Indeed, our key finding is that autistic persons do not monitor the value of their social environment to the same extent as that they do for their nonsocial environment. Importantly, the absence of covert face valuation was not related to autistic participants looking elsewhere or paying no attention. Their attention was driven to faces by the instruction to judge the age of the person, which they did with normal accuracy. Thus, the absence of covert face valuation was observed here, thanks to fMRI, while the behavior was kept constant across groups. It was neither due to a difference in the variance of likeability ratings: while autistic participants globally used a smaller portion of the likeability rating scale, this difference was even more pronounced with objects than with faces. Also, the absence of covert neural value signal does not mean that faces were globally less appreciated than objects: when explicitly asked, both groups tended to prefer objects to faces, but this tendency was less pronounced in autistic participants.

Thus, likeability may be seen as an attribute of social stimuli that autistic participants would not spontaneously estimate, together with other attributes such as mental states [22, 36, 37]. It could be an explanation for (but not a consequence of) these participants paying little attention to social stimuli in general [38]. However, some social information is normally processed in autistic persons: for instance, the goals of other people, when manifest in their behavior, can influence their own preferences for objects, as we have previously demonstrated [39, 40]. Taken together with the present findings, these previous results suggest that their interest in objects enable autistic persons to use social information to make inferences about their value (as an object that is pursued by another agent must be pleasant).

It has been suggested that faces are special stimuli for neurotypical persons but not for autistic persons [41]. Our findings support the opposite view: face and object neural value signals were more dissimilar here in autistic participants than in controls. The specific deficit observed in covert face valuation might relate to a special way to process faces, as documented in several studies [41–43]. It is possible that the concurrent orthogonal task was more demanding for autistic participants, which could prevent spontaneous face valuation. However, there was no indication in the behavior that estimating an age, which is rather an objective attribute of faces, was more difficult for autistic participants. Also, there is no evidence that automatic face valuation is sensitive to dual tasking (at least in control participants), here or in previous studies [23], as neural value signals were found similar under explicit and orthogonal instructions. It was on the contrary for objects that we noticed a decrease in neural value signals in the orthogonal task, compared to the valuation task. This could denote an interference with the concurrent size estimation task, or simply a lack of interest in (at least some) control participants for (at least some) of the objects presented in the task.

When making an overt face valuation, autistic participants seem to use the same brain areas as control participants. There was no indication, in their behavior or brain activity, that likeability ratings were computed in a different way compared to controls. It remains nonetheless possible that they base their valuation on different features. For instance, it has been shown that trustworthiness has a lesser impact on likeability ratings when social motivation is diminished [44]. This putative difference in the construction of face values could perhaps be tracked in other brain regions, which would provide the BVS with specific features. This is beyond our reach, because we did not systematically manipulate face features in our set of stimuli. Yet it may be linked to our results if we assume that the atypical features grounding face valuation in autistic persons fail to activate automatic processing by the BVS. Further research is needed to investigate the peculiarities of (explicit) face valuation in autistic persons.

Although it provides an interesting proof of concept, our study suffers from a number of limitations. A first limitation is that it relies on a small, mostly male sample of autistic persons without intellectual deficiency and with fluent language. Beyond the question of statistical power, our sample does not represent the developmental and symptomatic diversity of autism and we do not claim that it should be generalized to all autistic persons. On the contrary, it is quite plausible that our findings could be specific to a sub-sample of persons with autism. For example, previous findings suggested that neural responsivity to social rewards in autistic persons might depend on gender [45, 46]. However, due to our small number of participants, any attempt to go any further (in terms of gender, age, symptoms, or any other dimension) would be pure speculation at this stage. For a more comprehensive view of how autistic persons monitor their social environment, it would be necessary to assess larger clinical populations and to include other tests of social cognition in the same participants. Yet, the ability to undergo cognitive tests in a scanner might be a limitation inherent to this approach, as some autistic persons may not be able to perform some of the tests or experience distress linked to the noise of fMRI. A second limitation is that we did not use eye-tracking to monitor gaze fixation, such that we cannot formerly rule out a difference in the visual exploration of faces. Finally, we want to emphasize that the overt/covert (or explicit/implicit) distinction does not coincide with the conscious/unconscious distinction [47, 48]. Indeed, all pictures presented during the task were consciously perceived and we have no way to assess whether their value was consciously represented in participants’ minds during orthogonal tasks. The valuation was covert (implicit) in the sense that valuation signals were recorded in the brain even if the behavioral response was independent from valuation processes.

To conclude, fMRI enabled us to reveal an absence of covert face valuation in autism. This is showing a case, not so frequent in our opinion, where fMRI can tell us something about cognition that could not be inferred from the behavior. While this deficit might represent an important insight into social motivation in autism, further studies are needed to explore (1) how it relates to other aspects of the behavioral and neuro-cognitive phenotype and (2) the role it plays in the development of attention orientation and social expertize during childhood. To our knowledge, this is the first demonstration of a dissociation between explicit and implicit BVS processing in a neurodevelopmental condition. The logic could be equally applied to other domains and psychiatric disorders, which may show the same or a reverse dissociation. For instance, the BVS of patients with schizophrenia or depression, two disorders in which motivation disorders are observed [49, 50], might function differently when engaged spontaneously versus following explicit instructions—using different features and providing different values, or generating no value at all.

## Supplementary information

Supplementary Figure 1

## Footnotes

1 There is no consensus on how to designate people with autism. The different formulations, recommended by different groups or organizations, emphasize either the individual (person), an aspect of the identity (autism), or the pathological nature of the condition (disorder). In this article, we use the expressions “autistic person” for individuals and “autism spectrum” (AS).
