# Supplementary material for: Absence of covert face valuation in Autism
Source: Transl Psychiatry. 2021 Sep 7;11:463. doi: 10.1038/s41398-021-01551-z (PMC8423803; doi:10.1038/s41398-021-01551-z)

Supplementary Fig. 1 Neural confidence signals

a. Whole-brain analysis

Valuation task

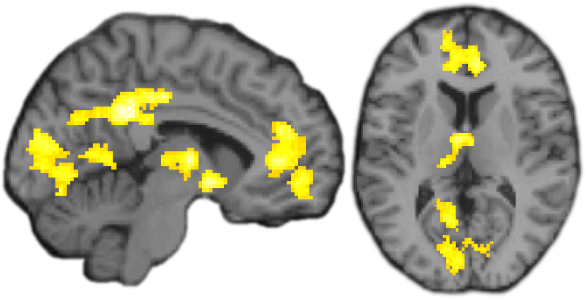

Valuation vs. Orthogonal Task

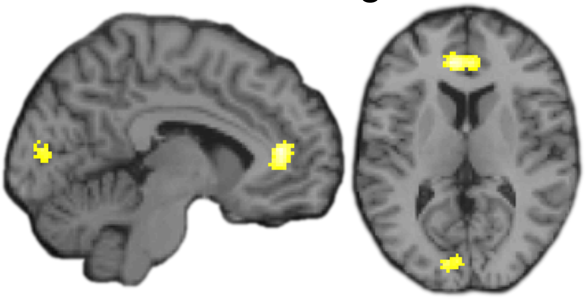

b. ROI analysis (vmPFC)

Valuation task

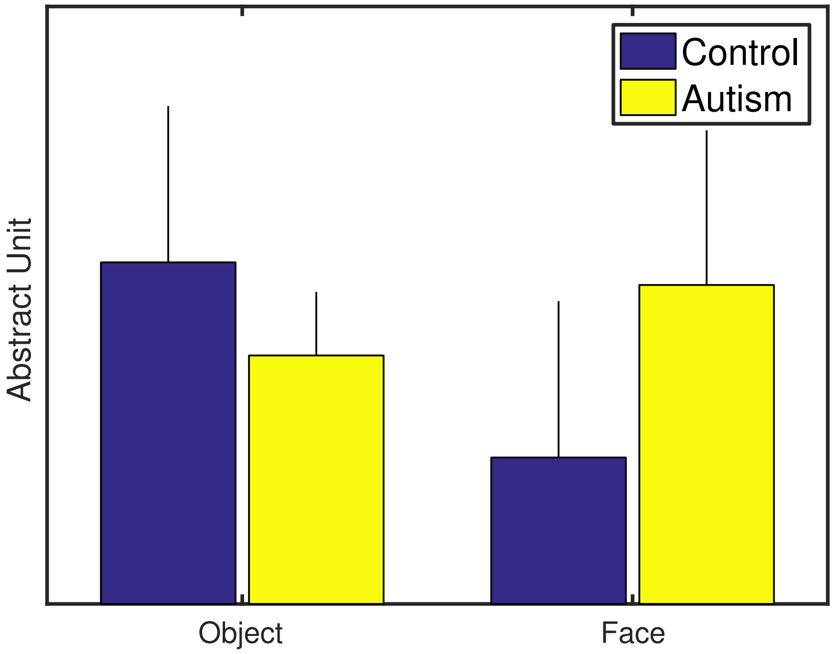

Supplement: Supplementary file 1 — Supplementary Figure 1 [file 41398_2021_1551_MOESM1_ESM.pdf]
